# Z-number network data envelopment analysis approach: A case study on the Iranian insurance industry

**DOI:** 10.1371/journal.pone.0306876

**Published:** 2024-07-11

**Authors:** Fatemeh Sadat Seyed Esmaeili, Emran Mohammadi

**Affiliations:** School of Industrial Engineering, Iran University of Science and Technology, Tehran, Iran; Azad University, ISLAMIC REPUBLIC OF IRAN

## Abstract

The main aim of this research is to present an innovative method known as fuzzy network data envelopment analysis (FNDEA) in order to assess the performance of network decision-making units (DMUs) that possess a two-stage structure while taking into account the uncertainty of data. To attain this goal, we utilize various methodologies including the non-cooperative game (leader-follower) NDEA method, the concept of Z-number, credibility theory, and chance-constrained programming (CCP) to develop a model for the fuzzy NDEA approach. The FNDEA approach offers several advantages, such as the linearity of the presented FNDEA models, the ability to rank two-stage DMUs in situations of ambiguity, the provision of a unique efficiency decomposition method in an uncertain environment, and the capability to handle Z-information. To demonstrate the applicability and effectiveness of the proposed approach, we implement the Z-number network data envelopment analysis (ZNDEA) approach in assessing the performance of Iranian private insurance companies. The results of this implementation reveal that the proposed ZNDEA method is suitable and effective for measuring and ranking insurance companies in situations where data ambiguity is present.

## 1. Introduction

Data envelopment analysis (DEA) is one of the popular and most widely used approaches in the field of performance evaluation. In DEA the concepts of relative efficiency (RE) and production possibility set (PPS) are used to assess the performance of homogeneous decision-making units (DMUs) to categorize the DMUs into efficient and inefficient groups [1–9]. The DEA technique is capable to provide a benchmark for inefficient DMUs where some extended DEA models can be utilized for sensitivity analysis, congestion estimation, and DMUs’ ranking [10–17].

It is important to note that traditional DEA models can only evaluate the performance of single-stage DMUs with a set of inputs and outputs. However, in mutual funds, supply chains, insurance companies, bank branches, manufacturing systems, and other real-world problems the DMUs have a network structure and there are different sub-units within each DMU [18–22]. In fact, these systems more complex than just having initial inputs and final outputs.

In such circumstances, there is a need for more novel approaches of DEA thecniques like network data envelopment analysis (NDEA) [23–28]. The network DEA approach measures the performance of DMUs by considering the internal structure of stages and sub-units of the DMUs as well as considering the internal relationships between sub-processes [29–33]. Using this method helps to identify and improve sub-units with undesirable performance.

Another important point in measuring the performance of network DMUs is uncertainty of data [34–37]. In numerous real-world scenarios and situations, data often lack precision and are uncertain. Hence, there is a need to develop a new NDEA approach that can evaluate the performance of DMUs with a network structure while considering data uncertainty. It is worth noting that various uncertain programming approaches, including stochastic optimization (SO), fuzzy optimization (FO), and robust optimization (RO), can be utilized depending on the type and nature of data uncertainty [38–43].

Fuzzy network data envelopment analysis (FNDEA) is an innovative method that integrates fuzzy mathematical programming and network structures to effectively manage imprecise and ambiguous data [44–50]. FNDEA considers the uncertainty and vagueness associated with the input and output variables and incorporates them into the efficiency measurement process. By applying FNDEA, decision-makers can identify the strengths and weaknesses of each DMU more effectively in network structure. As a results, FNDEA is an exceedingly valuable approach when it comes to measuring efficiency and determining the ranking of DMUs that possess network structures, especially in situations where the data is imprecise or vague. It provides a more realistic evaluation by considering the uncertainty and vagueness associated with the data, leading to better decision-making in real-world applications.

The goal of this research is to present a novel approach for fuzzy network data envelopment analysis in order to evaluate the performance of two-stage DMUs in situations characterized by deep ambiguity and a fuzzy environment. Accordingly, for handling deep ambiguity, the Z-number concept is employed. Additionally, for dealing with uncertainty in fuzzy chance-constraints of NDEA model, the credibility approach is utilized. Finally, the applicability and efficacy of the proposed Z-number network data envelopment analysis (ZNDEA) approach is demonstrated by evaluating the performance and ranking of 14 private insurance companies in Iran. The main contributions of the current research can be summarized as follows:

- A novel fuzzy NDEA approach is proposed using Z-number theory, credibility measure, fuzzy optimization, and chance-constrained programming.
- A leader-follower game method is applied for modeling of NDEA approach.
- The presented ZNDEA approach is capable to be used under Z-information.
- The ZNDEA method can be utilized to rank two-stage DMUs effectively.
- The literature on Z-number DEA is comprehensively reviewed and analyzed.
- The ZNDEA approach is proposed in a linear programming form.
- Efficiency decomposition is unique based on proposed ZNDEA method.
- The suggested method is utilized to assess the performance of the insurance industry.

The following sections of this paper are structured as follows. In Section 2, we will provide an introduction to the literature review and classification of Z-number DEA studies. Section 3 will delve into the preliminaries and theoretical background of the research, encompassing the network DEA modeling based on the leader-follower method, Z-number theory, and credibility theory. Moving on to Section 4, we will present the innovative Z-number network DEA approach. Subsequently, in Section 5, we will apply the proposed ZNDEA approach to a real-life case study involving private Iranian insurance companies, followed by an evaluation of the results. Lastly, Section 6 will encompass the conclusions drawn from the study as well as future research directions.

## 2. Literature review

In this section, an extensive examination and evaluation of previous research conducted on Z-number DEA studies will be introduced. Accordingly, the classification of Z-number DEA studies is illustrated in Table 1 by considering three characteristics, including the basic DEA model, the structure of DMU (black-box structure (BBS) or network structure (NS)), and the real-life applications of ZNDEA. The details regarding this research have also been outlined in the final row of Table 1.

**Table 1:** Data envelopment analysis and Z-number theory: A comprehensive review.

Year	Research	DEA Model	Application & Data Set	DMU Structure
2015	Sahrom & Dom [51]	CCR [1]	Bridge Structures Risk Assessment	BBS
2016	Azadeh & Kokabi [52]	CCR	Project Portfolio Selection	BBS
2016	Sadi-Nezhad & Sotoudeh-Anvari [53]	CCR	Numerical Example	BBS
2016	Sotoudeh-Anvari et al. [54]	CCR	Numerical Example	BBS
2017	Tohidifard et al. [55]	CCR	Emergency Departments	BBS
2018	Sadeghsa et al. [56]	CCR	Petrochemical Plants	BBS
2019	Akbarian Saravi et al. [57]	CCR	Biomass Plants	BBS
2020	Abbasi et al. [58]	CCR	Land Suitability Assessment	BBS
2020	Fakhari et al. [59]	CCR	Gas Power Plants Location Optimization	BBS
2020	Mohtashami & Ghiasvand [60]	ERM [2]	Banks & Financial Institutes in Stock Exchange	BBS
2021	Tavana et al. [61]	CCR	Supplier Evaluation & Selection in Health Care	BBS
2021	Yazdanparast et al. [62]	CCR	Supply Chain Resilience Assessment	BBS
2022	Kuchta & Gładysz [63]	CCR	Project Portfolio Selection	BBS
2022	RezaHoseini et al. [64]	CCR	Sustainable Project Selection	BBS
2023	Nazari-Shirkouhi et al. [65]	CCR	Resilient Supplier Selection	BBS
2023	Peykani et al. [66]	CCR	Numerical Example	BBS
2023	Zhang & Li [67]	Cross-Efficiency	Location Problem	BBS
The Current Research		Two-Stage DEA	Insurance Industry	NS

Based on the comprehensive analysis of existing literature and referring to the information presented in Table 1, it is clear that the Z-number DEA approach has not been proposed for a network structure so far. Hence, this domain presents prospective avenues for future exploration and untapped research possibilities. Consequently, the current research proposes a new fuzzy network DEA approach based on the leader-follower method using Z-number theory and credibility measure to appraise the performance of network DMUs with a two-stage structure under fuzzy data and linguistic variables.

## 3. Preliminaries

In this section, an overview of the theoretical foundation of the research including network DEA modeling based on non-cooperative game (leader-follower) method, definitions and relations of Z-number theory, and credibility measure will be proposed.

### 3.1. Two-stage DEA: The leader-follower approach

**Fig 1:**
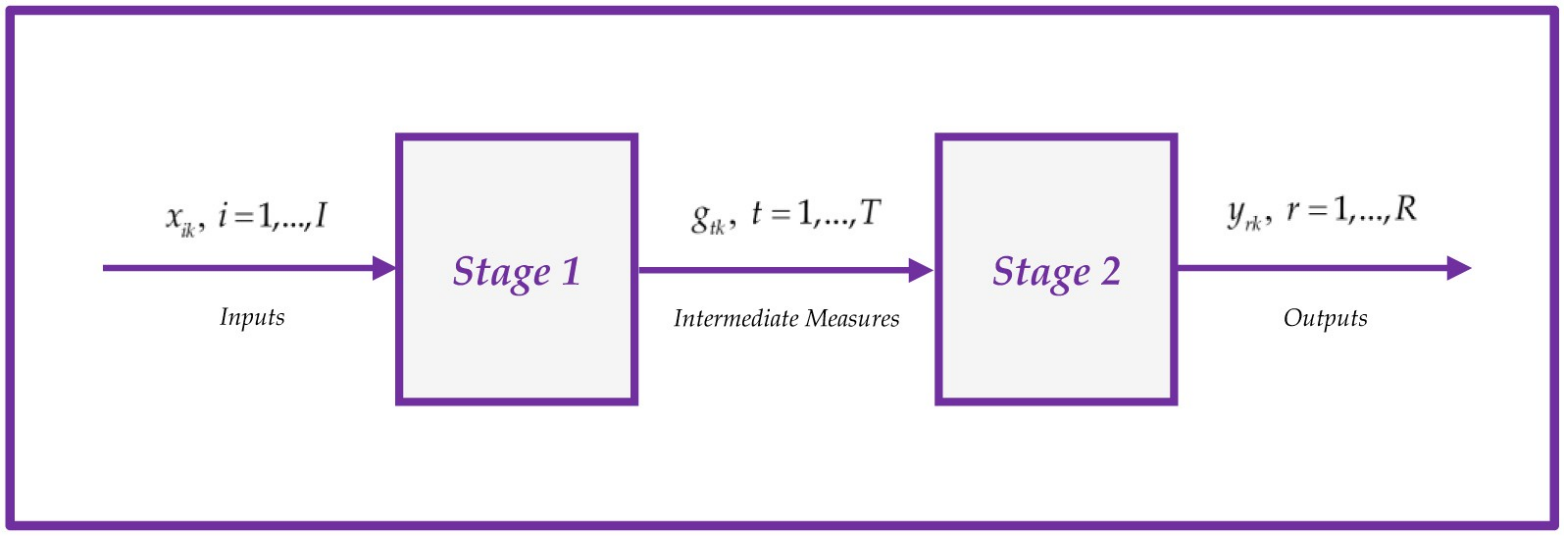
Two-stage network structure.

In this study, we define *x*_*ik*_ (*i* = 1, …, *I*) as the input variables at the initial stage, *g*_*tk*_ (*t* = 1, …, *T*) as the intermediate variables that connect the two stages, and *y*_*rk*_ (*r* = 1, …, *R*) as the output variables at the second stage for DMU_*k*_ (*k* = 1, …, *K*). Furthermore, we assign non-negative weights *v*_*i*_ (*i* = 1, …, *I*), *w*_*t*_ (*t* = 1, …, *T*), and *u*_*r*_ (*r* = 1, …, *R*) to the inputs, intermediate measures, and outputs respectively. The structure of the current study is visually depicted in Fig 1, showcasing the two-stage process:

The leader-follower NDEA approach is a widely recognized and practical technique for modeling network DEA problems [68–73]. This approach assumes that one of the stages or processes holds greater significance and is designated as the leader. Consequently, the leader’s stage efficiency is initially evaluated, and then the efficiency of the remaining stages or follower’s stages is determined based on the optimal solution value of the leader’s stage. Referring to Fig 1, if we consider the first stage as the more important one, the efficiency of the first stage for the evaluated *DMU*_*p*_ is calculated using Model (1):

$$\Theta_{p}=Max\;\frac{\sum_{t=1}^{T}g_{tp}w_{t}}{\sum_{i=1}^{I}x_{ip}v_{i}}$$ (1)

$$S.t.\;\frac{\sum_{t=1}^{T}g_{tk}w_{t}}{\sum_{i=1}^{I}x_{ik}v_{i}}\leq 1,\;\forall k=1,\ldots ,K$$

$$v_{i},\;w_{t}\geq \varepsilon ,\;\forall \;i=1,\ldots ,I,\;t=1,\ldots ,T$$

It should be explained that the optimal value of the objective function in Model (1) is the first stage efficiency score of DMU_*p*_ and is always $0<\Theta_{p}^{*}\leq 1$. Also, if the efficiency score being one, DMU will be efficient in the first stage, and if the efficiency score being less than one, DMU will be inefficient from first stage viewpoint. Given that Model (1) is a linear fractional program (LFP), we can employ the Charnes & Cooper transformation [74] to convert this particular model into Model (2) [68]:

$$\Theta_{p}=Max\;\sum_{t=1}^{T}g_{tp}w_{t}$$ (2)

$$S.t.\;\sum_{i=1}^{I}x_{ip}v_{i}=1$$

$$\sum_{t=1}^{T}g_{tk}w_{t}-\sum_{i=1}^{I}x_{ik}v_{i}\leq 0,\;\forall \;k=1,\ldots ,K$$

$$v_{i},\;w_{t}\geq \varepsilon ,\;\forall \;i=1,\ldots ,I,\;t=1,\ldots ,T$$

Next, we proceed to assess the effectiveness of the second phase utilizing Model (3), while optimizing the parameter $\Theta_{p}^{*}$ based on Model (1) [68]:

$$\Phi_{p}=Max\;\frac{\sum_{r=1}^{R}y_{rp}u_{r}}{\sum_{t=1}^{T}g_{tp}w_{t}}$$ (3)

$$S.t.\;\frac{\sum_{r=1}^{R}y_{rk}u_{r}}{\sum_{t=1}^{T}g_{tk}w_{t}}\leq 1,\;\forall k=1,\ldots ,K$$

$$\frac{\sum_{t=1}^{T}g_{tk}w_{t}}{\sum_{i=1}^{I}x_{ik}v_{i}}\leq 1,\;\forall k=1,\ldots ,K$$

$$\frac{\sum_{t=1}^{T}g_{tp}w_{t}}{\sum_{i=1}^{I}x_{ip}v_{i}}=\Theta_{p}^{*}$$

$$v_{i},\;w_{t},\;u_{r}\geq \varepsilon ,\;\forall \;i=1,\ldots ,I,\;t=1,\ldots ,T,\;r=1,\ldots ,R$$

Given that the first stage is considered as a leader, Model (3) will be solved to discover a collection of multipliers that yield the highest possible efficiency score for the second stage, while ensuring that the efficiency score of the first stage is preserved. Note that the optimal value of the objective function in Model (3) is the second stage efficiency score of DMU_*p*_ and is always $0<\Phi_{p}^{*}\leq 1$. Also, if the efficiency score being one, DMU will be efficient in the second stage, and if the efficiency score being less than one, DMU will be inefficient from second stage viewpoint. As evident in Model (3), this model is a LFP which can be transformed to linear program as Model (4):

$$\Phi_{p}=Max\;\sum_{r=1}^{R}y_{rp}u_{r}$$ (4)

$$S.t.\;\sum_{t=1}^{T}g_{tp}w_{t}=1$$

$$\sum_{r=1}^{R}y_{rk}u_{r}-\sum_{t=1}^{T}g_{tk}w_{t}\leq 0,\;\forall \;k=1,\ldots ,K$$

$$\sum_{t=1}^{T}g_{tk}w_{t}-\sum_{i=1}^{I}x_{ik}v_{i}\leq 0,\;\forall \;k=1,\ldots ,K$$

$$\sum_{t=1}^{T}g_{tp}w_{t}-\Theta_{p}^{*}\sum_{i=1}^{I}x_{ip}v_{i}=0$$

$$v_{i},\;w_{t},\;u_{r}\geq \varepsilon ,\;\forall \;i=1,\ldots ,I,\;t=1,\ldots ,T,\;r=1,\ldots ,R$$

Finally, the overall efficiency of DMU_*p*_ is the product of the efficiencies of the two stages. As a result, the overall efficiency score of DMU_*p*_ is calculated according to Eq (5) as follows:

$$\Psi_{p}^{*}=\Theta_{p}^{*}\;\Phi_{p}^{*}$$ (5)

In an alternate scenario, if it is believed that the second stage holds greater significance, a similar approach can be taken. Initially, the efficiency score of the second stage is computed, followed by the estimation of the efficiency score for the first stage and the entire two-stage system, respectively.

### 3.2. Z-number theory

Z-number theory, initially introduced by Zadeh [75], encompasses the fundamental principles that underpin the computation of numbers with inherent uncertainty. The notion of Z-number serves as a cornerstone in this theory, enabling the manipulation of numerical values that lack complete reliability. It is crucial to elucidate that a Z-number comprises two distinct components, referred to as $\tilde{Z}=(\tilde{\alpha },\;\tilde{\beta })$, which aid in estimating the variable *λ*. The first component, denoted as $\tilde{\alpha }$, imposes restriction on the potential values that *λ* can assume, while the second component, represented by $\tilde{\beta }$, quantifies the reliability associated with the first component [52]. Nowadays, Z-number theory is one of the popular and applicable method for description of uncertain phenomena in real-word problems such as financial market [76]. Please note that due to computational complexity, Z-number is almost converted to classical fuzzy number (CFN) or crisp number in the literature. Kang et al. [77] suggested a popular procedure during three steps for converting Z-number to CFN as follows:

**Step 1:** Converting the reliability component $\tilde{\beta }$ into a crisp number using Eq (6), where ∫ denotes an algebraic integration:
$$\delta =\frac{\int \lambda \mu_{\tilde{\beta }}(\lambda )d\lambda }{\int \mu_{\tilde{\beta }}(\lambda )d\lambda }$$ (6)
  If assumed that $\tilde{\beta }$ is a triangular fuzzy variable $\tilde{\beta }(\beta^{\left(1\right)},\beta^{\left(2\right)},\beta^{\left(3\right)})$, Eq (6) becomes as Eq (7):
$$\delta =\frac{\beta^{\left(1\right)}+\beta^{\left(2\right)}+\beta^{\left(3\right)}}{3}$$ (7)
**Step 2:** Adding weight of the reliability component $\tilde{\beta }$ to the restriction component is. Accordingly, the weighted Z-number can be expressed by Eq (8):
$${\tilde{Z}}^{\delta }=\left\{\lambda ,\;\mu_{{\tilde{\alpha }}^{\delta }}(\lambda )|\mu_{{\tilde{\alpha }}^{\delta }}(\lambda )=\delta \;\mu_{\tilde{\alpha }}(\lambda ),\;\lambda \in \left[0,\;1\right]\right\}$$ (8)
**Step 3:** Converting the weighted Z-number to CFN by multiplying $\sqrt{\delta }$. If assumed that $\tilde{\alpha }$ is a trapezoidal fuzzy variable $\tilde{\alpha }(\alpha^{\left(1\right)},\alpha^{\left(2\right)},\alpha^{\left(3\right)},\alpha^{\left(4\right)})$, final CFN is calculated as follows:
$${\tilde{Z}}^{\prime }=\sqrt{\delta }\times {\tilde{\alpha }}^{\delta }=(\sqrt{\delta }\times \alpha^{\left(1\right)},\sqrt{\delta }\times \alpha^{\left(2\right)},\sqrt{\delta }\times \alpha^{\left(3\right)},\sqrt{\delta }\times \alpha^{\left(4\right)})$$ (9)

It should be noted that in this paper, Z-numbers using approach of Kang et al. [77], will be converted to CFN.

### 3.3. Credibility measure

Based on Liu and Liu [78], the credibility (*Cr*) measure of {*G*} is defined on the possibility space (Δ, *P*(Δ), *Pos*) as the average of its possibility (*Pos*) and necessity (*Nec*) measures as follows:

$$Cr\{G\}=0.5\left(Pos\{G\}+Nec\{G\}\right)$$ (10)

The expected value (EV) of the trapezoidal fuzzy variable $\tilde{\varpi }(\varpi^{\left(1\right)},\varpi^{\left(2\right)},\varpi^{\left(3\right)},\varpi^{\left(4\right)})$ using the credibility measure is defined as Eq (11):

$$E^{Cr}\left[\tilde{\varpi }\right]=\int_{0}^{+\infty }Cr\left\{\tilde{\varpi }\geq \varsigma \right\}d\varsigma -\int_{-\infty }^{0}Cr\left\{\tilde{\varpi }\leq \varsigma \right\}d\varsigma =\left(\frac{\varpi^{\left(1\right)}+\varpi^{\left(2\right)}+\varpi^{\left(3\right)}+\varpi^{\left(4\right)}}{4}\right)$$ (11)

Let $\tilde{\varpi }$ be a trapezoidal fuzzy variable on the possibility space (Δ, *P*(Δ), *Pos*) and *ϛ* be a crisp number. The credibility measure of fuzzy events $\left\{\tilde{\varpi }\leq \varsigma \right\}$ and $\left\{\tilde{\varpi }\geq \varsigma \right\}$ are shown in Eqs (12) and (13), respectively:

$$Cr\left\{\tilde{\varpi }\leq \varsigma \right\}=\left\{\begin{array}{ll} 0, & \varsigma \in \left(-\infty ,\varpi^{\left(1\right)}\right],\\ \frac{\varsigma -\varpi^{\left(1\right)}}{2\left(\varpi^{\left(2\right)}-\varpi^{\left(1\right)}\right)}, & \varsigma \in \left(\varpi^{\left(1\right)},\varpi^{\left(2\right)}\right],\\ 0.5, & \varsigma \in \left(\varpi^{\left(2\right)},\varpi^{\left(3\right)}\right],\\ \frac{\varsigma -2\varpi^{\left(3\right)}+\varpi^{\left(4\right)}}{2\left(\varpi^{\left(4\right)}-\varpi^{\left(3\right)}\right)}, & \varsigma \in \left(\varpi^{\left(3\right)},\varpi^{\left(4\right)}\right],\\ 1, & \varsigma \in \left(\varpi^{\left(4\right)},+\infty \right]. \end{array}\right.$$ (12)

$$Cr\left\{\tilde{\varpi }\geq \varsigma \right\}=\left\{\begin{array}{ll} 1, & \varsigma \in \left(-\infty ,\varpi^{\left(1\right)}\right],\\ \frac{2\varpi^{\left(2\right)}-\varpi^{\left(1\right)}-\varsigma }{2\left(\varpi^{\left(2\right)}-\varpi^{\left(1\right)}\right)}, & \varsigma \in \left(\varpi^{\left(1\right)},\varpi^{\left(2\right)}\right],\\ 0.5, & \varsigma \in \left(\varpi^{\left(2\right)},\varpi^{\left(3\right)}\right],\\ \frac{\varpi^{\left(4\right)}-\varsigma }{2\left(\varpi^{\left(4\right)}-\varpi^{\left(3\right)}\right)}, & \varsigma \in \left(\varpi^{\left(3\right)},\varpi^{\left(4\right)}\right],\\ 0, & \varsigma \in \left(\varpi^{\left(4\right)},+\infty \right]. \end{array}\right.$$ (13)

Based on the credibility measure, the fuzzy chance constraints can be transformed into crisp equivalents at the desired confidence level *ξ* as follows [79–81]:

$$Cr\left\{\tilde{\varpi }\leq \varsigma \right\}\geq \xi \Leftrightarrow \left\{\begin{array}{ll} \left(2-2\xi \right)\;\varpi^{\left(3\right)}+\left(2\xi -1\right)\;\varpi^{\left(4\right)}\leq \varsigma & if\;\xi >0.5;\\ \left(1-2\xi \right)\;\varpi^{\left(1\right)}+\left(2\xi \right)\;\varpi^{\left(2\right)}\leq \varsigma & if\;\xi \leq 0.5. \end{array}\right.$$ (14)

$$Cr\left\{\tilde{\varpi }\geq \varsigma \right\}\geq \xi \;\Leftrightarrow \;\left\{\begin{array}{ll} \left(2\xi -1\right)\;\varpi^{\left(1\right)}+\left(2-2\xi \right)\;\varpi^{\left(2\right)}\geq \varsigma & if\;\xi >0.5;\\ \left(2\xi \right)\;\varpi^{\left(3\right)}+\left(1-2\xi \right)\;\varpi^{\left(4\right)}\geq \varsigma & if\;\xi \leq 0.5. \end{array}\right.$$ (15)

As it can be seen in Eqs (14) and (15), an equivalent crisp of fuzzy chance constraints for conditions *ξ* > 0.5 and *ξ* ≤ 0.5 are different. In this study, the condition of *ξ* > 0.5 is considered for proposing equivalent crisp of fuzzy chance constraints.

## 4. Z-number network DEA approach

The goal of this section is to propose a novel fuzzy NDEA approach for performance evaluation of two-stage DMUs that is capable to be used in the presence of Z-information. To reach this goal, the leader-follower method (first stage is considered as a leader), Z-number theory, and credibility measure will be employed. Accordingly, suppose that all data are represented by Z-numbers ${\tilde{x}}^{\tilde{Z}}=({\tilde{x}}^{\tilde{\alpha }},\;{\tilde{x}}^{\tilde{\beta }})$, ${\tilde{g}}^{\tilde{Z}}=({\tilde{g}}^{\tilde{\alpha }},\;{\tilde{g}}^{\tilde{\beta }})$, and ${\tilde{y}}^{\tilde{Z}}=({\tilde{y}}^{\tilde{\alpha }},\;{\tilde{y}}^{\tilde{\beta }})$. It should be note that first component $\tilde{\alpha }$ and second component $\tilde{\beta }$ of all Z-numbers have trapezoidal fuzzy distribution and triangular fuzzy distribution, respectively. Additionally, taking into account the ambiguity associated with all the available data, Models (2) and (4) can be converted to Models (16) and (17) correspondingly:

$${\tilde{\Theta }}_{p}=Max\;\Omega$$ (16)

$$S.t.\;\sum_{t=1}^{T}{\tilde{g}}_{tp}^{Z}w_{t}\geq \Omega$$

$$\sum_{i=1}^{I}{\tilde{x}}_{ip}^{Z}\;v_{i}\leq 1$$

$$\sum_{t=1}^{T}{\tilde{g}}_{tk}^{Z}\;w_{t}-\sum_{i=1}^{I}{\tilde{x}}_{ik}^{Z}\;v_{i}\leq 0,\;\forall \;k=1,\ldots ,K$$

$$v_{i},\;w_{t}\geq \varepsilon ,\;\forall \;i=1,\ldots ,I,\;t=1,\ldots ,T$$

$${\tilde{\Phi }}_{p}=Max\;ϒ$$ (17)

$$S.t.\;\sum_{r=1}^{R}{\tilde{y}}_{rp}^{Z}\;u_{r}\geq ϒ$$

$$\sum_{t=1}^{T}{\tilde{g}}_{tp}^{Z}\;w_{t}\leq 1$$

$$\sum_{r=1}^{R}{\tilde{y}}_{rk}^{Z}\;u_{r}-\sum_{t=1}^{T}{\tilde{g}}_{tk}^{Z}\;w_{t}\leq 0,\;\forall \;k=1,\ldots ,K$$

$$\sum_{t=1}^{T}{\tilde{g}}_{tk}^{Z}\;w_{t}-\sum_{i=1}^{I}{\tilde{x}}_{ik}^{Z}\;v_{i}\leq 0,\;\forall \;k=1,\ldots ,K$$

$$\sum_{t=1}^{T}{\tilde{g}}_{tp}^{Z}\;w_{t}-{\tilde{\Theta }}_{p}^{*}\;\sum_{i=1}^{I}{\tilde{x}}_{ip}^{Z}\;v_{i}\geq 0$$

$$v_{i},\;w_{t},\;u_{r}\geq \varepsilon ,\;\forall \;i=1,\ldots ,I,\;t=1,\ldots ,T,\;r=1,\ldots ,R$$

It is important to take note that modifications have been made to the equality constraint and the objective function of Models (16) and (17). However, it should be emphasized that none of these alterations have any impact on the optimal solutions of these models (for more details see [79, 80]). Also Ω and Υ are considered as Ω(Ω^(1)^, Ω^(2)^, Ω^(3)^, Ω^(4)^) and Υ(Υ^(1)^, Υ^(2)^, Υ^(3)^, Υ^(4)^), respectively. Now, all Z-numbers ${\tilde{x}}^{\tilde{Z}}=({\tilde{x}}^{\tilde{\alpha }},\;{\tilde{x}}^{\tilde{\beta }})$, ${\tilde{g}}^{\tilde{Z}}=({\tilde{g}}^{\tilde{\alpha }},\;{\tilde{g}}^{\tilde{\beta }})$, and ${\tilde{y}}^{\tilde{Z}}=({\tilde{y}}^{\tilde{\alpha }},\;{\tilde{y}}^{\tilde{\beta }})$ are converted to the classic fuzzy numbers ${\tilde{x}}_{ik}\left(x_{ik}^{\left(1\right)},\;x_{ik}^{\left(2\right)},\;x_{ik}^{\left(3\right)},\;x_{ik}^{\left(4\right)}\right)$, ${\tilde{g}}_{tk}\left(g_{tk}^{\left(1\right)},\;g_{tk}^{\left(2\right)},\;g_{tk}^{\left(3\right)},\;g_{tk}^{\left(4\right)}\right)$, and ${\tilde{y}}_{rk}\left(y_{rk}^{\left(1\right)},\;y_{rk}^{\left(2\right)},\;y_{rk}^{\left(3\right)},\;y_{rk}^{\left(4\right)}\right)$ in which $x_{ik}^{\left(1\right)}<x_{ik}^{\left(2\right)}<x_{ik}^{\left(3\right)}<x_{ik}^{\left(4\right)}$, $g_{tk}^{\left(1\right)}<g_{tk}^{\left(2\right)}<g_{tk}^{\left(3\right)}<g_{tk}^{\left(4\right)}$, and $y_{rk}^{\left(1\right)}<y_{rk}^{\left(2\right)}<y_{rk}^{\left(3\right)}<y_{rk}^{\left(4\right)}$. Then, the credibility measure and chance-constrained programming will be applied to deal with fuzzy chance constrains as follows:

$${\tilde{\Theta }}_{p}=Max\;\Omega$$ (18)

$$S.t.\;Cr\left\{\sum_{t=1}^{T}{\tilde{g}}_{tp}\;w_{t}\geq \Omega \right\}\geq \xi$$

$$Cr\left\{\sum_{i=1}^{I}{\tilde{x}}_{ip}\;v_{i}\leq 1\right\}\geq \xi$$

$$Cr\left\{\sum_{t=1}^{T}{\tilde{g}}_{tk}w_{t}-\sum_{i=1}^{I}{\tilde{x}}_{ik}v_{i}\leq 0\right\}\geq \xi ,\;\forall \;k=1,\ldots ,K$$

$$v_{i},\;w_{t}\geq \varepsilon ,\;\forall \;i=1,\ldots ,I,\;t=1,\ldots ,T$$

$${\tilde{\Phi }}_{p}=Max\;ϒ$$ (19)

$$S.t.\;Cr\left\{\sum_{r=1}^{R}{\tilde{y}}_{rp}u_{r}\geq ϒ\right\}\geq \xi$$

$$Cr\left\{\sum_{t=1}^{T}{\tilde{g}}_{tp}w_{t}\leq 1\right\}\geq \xi$$

$$Cr\left\{\sum_{r=1}^{R}{\tilde{y}}_{rk}u_{r}-\sum_{t=1}^{T}{\tilde{g}}_{tk}w_{t}\leq 0\right\}\geq \xi ,\;\forall k=1,\ldots ,K$$

$$Cr\left\{\sum_{t=1}^{T}{\tilde{g}}_{tk}w_{t}-\sum_{i=1}^{I}{\tilde{x}}_{ik}v_{i}\leq 0\right\}\geq \xi ,\;\forall k=1,\ldots ,K$$

$$Cr\left\{\sum_{t=1}^{T}{\tilde{g}}_{tp}w_{t}-\Theta_{p}^{*}\sum_{i=1}^{I}{\tilde{x}}_{ip}v_{i}\geq 0\right\}\geq \xi$$

$$v_{i},\;w_{t},\;u_{r}\geq \varepsilon ,\;\forall \;i=1,\ldots ,I,\;t=1,\ldots ,T,\;r=1,\ldots ,R$$

Now, by using Eqs (14) and (15), an equivalent crisp forms of fuzzy chance constraints of Models (18) and (19) under specific confidence level *ξ* > 0.5, can be written as Models (20) and (21), respectively:

$$\Theta_{p}^{\xi }=Max\;\Omega$$ (20)

$$S.t.\;\sum_{t=1}^{T}\left(\left(2\xi -1\right)g_{tp}^{\left(1\right)}+\left(2-2\xi \right)g_{tp}^{\left(2\right)}\right)w_{t}\geq \Omega$$

$$\sum_{i=1}^{I}\left(\left(2-2\xi \right)x_{ip}^{\left(3\right)}+\left(2\xi -1\right)x_{ip}^{\left(4\right)}\right)v_{i}\leq 1$$

$$\sum_{t=1}^{T}\left(\left(2-2\xi \right)g_{tk}^{\left(3\right)}+\left(2\xi -1\right)g_{tk}^{\left(4\right)}\right)w_{t}-\sum_{i=1}^{I}\left(\left(2\xi -1\right)x_{ik}^{\left(1\right)}+\left(2-2\xi \right)x_{ik}^{\left(2\right)}\right)v_{i}\leq 0,\;\forall k=1,\ldots ,K$$

$$v_{i},\;w_{t}\geq \varepsilon ,\;\forall i=1,\ldots ,I,\;t=1,\ldots ,T$$

**Proposition 1:** For Model (20), there exists a feasible solution.

**Proof.** Suppose $\left(\bar{v},\;\bar{w},\;\bar{\Omega }\right)$ is the optimal solution of Model (20). Next, let the items be replaced in the following manner: ${\bar{v}}_{i}=\frac{1}{I\tau }(i=1,\ldots ,I)$, ${\bar{w}}_{t}=\varepsilon (t=1,\ldots ,T)$, $\bar{\Omega }=0$, $\left(\left(2\xi -1\right)g_{tp}^{\left(1\right)}+\left(2-2\xi \right)g_{tp}^{\left(2\right)}\right)=\mu_{t}$, $\left(\left(2-2\xi \right)x_{ip}^{\left(3\right)}+\left(2\xi -1\right)x_{ip}^{\left(4\right)}\right)=\tau_{i}$, $\left(\left(2-2\xi \right)g_{tk}^{\left(3\right)}+\left(2\xi -1\right)g_{tk}^{\left(4\right)}\right)=\rho_{t}$, and $\left(\left(2\xi -1\right)x_{ik}^{\left(1\right)}+\left(2-2\xi \right)x_{ik}^{\left(2\right)}\right)=\gamma_{i}$, where *τ* is calculated as *τ* = *Max* {*τ*_*i*_} > 0. Because all the inputs are not zero at the same time, then we have *τ* > 0. It should be explained that we have in the first constraint $\sum_{t=1}^{T}\left(\left(2\xi -1\right)g_{tp}^{\left(1\right)}+\left(2-2\xi \right)g_{tp}^{\left(2\right)}\right){\bar{w}}_{t}\geq \bar{\Omega }$, and we will have with replacement $\sum_{t=1}^{T}\mu_{t}\varepsilon \geq 0$, as a result, the first constraint is established. Also, we have in the second constraint $\sum_{i=1}^{I}\left(\left(2-2\xi \right)x_{ip}^{\left(3\right)}+\left(2\xi -1\right)x_{ip}^{\left(4\right)}\right){\bar{v}}_{i}$, and we will have with replacement ${\bar{v}}_{i}\tau_{i}\leq {\bar{v}}_{i}\tau =\frac{1}{I}$, that is $\sum_{i=1}^{I}{\bar{v}}_{i}\tau_{i}\leq \sum_{i=1}^{I}\frac{1}{I}=1$, as a result, the second constraint is established. Moreover, that we have in the third constraint $\sum_{t=1}^{T}\left(\left(2-2\xi \right)g_{tk}^{\left(3\right)}+\left(2\xi -1\right)g_{tk}^{\left(4\right)}\right){\bar{w}}_{t}-\sum_{i=1}^{I}\left(\left(2\xi -1\right)x_{ik}^{\left(1\right)}+\left(2-2\xi \right)x_{ik}^{\left(2\right)}\right){\bar{v}}_{i},\;\forall k=1,\ldots ,K$, and we will have with replacement $\sum_{t=1}^{T}\rho_{t}{\bar{w}}_{t}-\sum_{i=1}^{I}\gamma_{i}{\bar{v}}_{i}=\varepsilon .\sum_{t=1}^{T}\rho_{t}-\frac{1}{I\tau }\sum_{i=1}^{I}\gamma_{i}\leq 0$. Because *ε* is a non-Archimedean number and small enough, and also all *γ*_*i*_ are not zero, that is, $\frac{1}{I\tau }\sum_{i=1}^{I}\gamma_{i}>0$ then $\varepsilon .\sum_{t=1}^{T}\rho_{t}\leq \frac{1}{I\tau }\sum_{i=1}^{I}\gamma_{i}$, as a result, the third constraint is established. Accordinlgy, for Model (20), there exists a feasible solution.


$$\Phi_{p}^{\xi }=Max\;ϒ$$ (21)

$$S.t.\;\sum_{r=1}^{R}\left(\left(2\xi -1\right)y_{rp}^{\left(1\right)}+\left(2-2\xi \right)y_{rp}^{\left(2\right)}\right)u_{r}\geq ϒ$$

$$\sum_{t=1}^{T}\left(\left(2-2\xi \right)g_{tp}^{\left(3\right)}+\left(2\xi -1\right)g_{tp}^{\left(4\right)}\right)w_{t}\leq 1$$

$$\sum_{r=1}^{R}\left(\left(2-2\xi \right)y_{rk}^{\left(3\right)}+\left(2\xi -1\right)y_{rk}^{\left(4\right)}\right)u_{r}-\sum_{t=1}^{T}\left(\left(2\xi -1\right)g_{tk}^{\left(1\right)}+\left(2-2\xi \right)g_{tk}^{\left(2\right)}\right)w_{t}\leq 0,\;\forall k=1,\ldots ,K$$

$$\sum_{t=1}^{T}\left(\left(2-2\xi \right)g_{tk}^{\left(3\right)}+\left(2\xi -1\right)g_{tk}^{\left(4\right)}\right)w_{t}-\sum_{i=1}^{I}\left(\left(2\xi -1\right)x_{ik}^{\left(1\right)}+\left(2-2\xi \right)x_{ik}^{\left(2\right)}\right)v_{i}\leq 0,\;\forall k=1,\ldots ,K$$

$$\sum_{t=1}^{T}\left(\left(2\xi -1\right)g_{tp}^{\left(1\right)}+\left(2-2\xi \right)g_{tp}^{\left(2\right)}\right)w_{t}-\Theta_{p}^{\xi *}\sum_{i=1}^{I}\left(\left(2-2\xi \right)x_{ip}^{\left(3\right)}+\left(2\xi -1\right)x_{ip}^{\left(4\right)}\right)v_{i}\geq 0$$

$$v_{i},\;w_{t},\;u_{r}\geq \varepsilon ,\;\forall \;i=1,\ldots ,I,\;t=1,\ldots ,T,\;r=1,\ldots ,R$$

**Proposition 2:** For Model (21), there exists a feasible solution.

**Proof.** Suppose $\left(v^{*},\;w^{*},\;u^{*},\;{ϒ}^{*}\right)$ is the optimal solution of Model (21). Next, let the items be replaced in the following manner: $v_{i}=v_{t}^{*}$, $w_{t}=w_{t}^{*}$, *u*_*r*_ = *ε*, Υ = 0, $\left(\left(2\xi -1\right)y_{rp}^{\left(1\right)}+\left(2-2\xi \right)y_{rp}^{\left(2\right)}\right)=M_{r}$, $\left(\left(2-2\xi \right)g_{tk}^{\left(3\right)}+\left(2\xi -1\right)g_{tk}^{\left(4\right)}\right)=\rho_{t}$,$\left(\left(2-2\xi \right)y_{rk}^{\left(3\right)}+\left(2\xi -1\right)y_{rk}^{\left(4\right)}\right)=\lambda_{r}$, $\left(\left(2\xi -1\right)g_{tp}^{\left(1\right)}+\left(2-2\xi \right)g_{tp}^{\left(2\right)}\right)=\mu_{t}$, $\left(\left(2-2\xi \right)x_{ip}^{\left(3\right)}+\left(2\xi -1\right)x_{ip}^{\left(4\right)}\right)=\tau_{i}$, and $\left(\left(2\xi -1\right)x_{ik}^{\left(1\right)}+\left(2-2\xi \right)x_{ik}^{\left(2\right)}\right)=\gamma_{i}$. It should be explained that by replacing Υ = 0 in the first constraint, it is clear that the first constraint is established $\varepsilon \sum_{r=1}^{R}M_{r}\geq 0$. In the third constraint, since *u*_*r*_ = *ε* and *ε* are small enough and $\sum_{t=1}^{T}\mu_{t}w_{t}^{*}$ is positive, then $\sum_{r=1}^{R}\lambda_{r}u_{r}=\varepsilon \sum_{r=1}^{R}\lambda_{r}\leq \sum_{t=1}^{T}\mu_{t}w_{t}^{*}$, as a result, the third constraint is also established. From Model (20), we have $\sum_{t=1}^{T}\left(\left(2-2\xi \right)g_{tk}^{\left(3\right)}+\left(2\xi -1\right)g_{tk}^{\left(4\right)}\right)w_{t}-\sum_{i=1}^{I}\left(\left(2\xi -1\right)x_{ik}^{\left(1\right)}+\left(2-2\xi \right)x_{ik}^{\left(2\right)}\right)v_{i}\leq 0,\;\forall k=1,\ldots ,K$, and we will have with replacement $\sum_{t=1}^{T}\rho_{t}w_{t}\leq \sum_{i=1}^{I}\gamma_{i}v_{i}$, as a result, the fourth constraint is established. In the fifth constraint, since $\sum_{i=1}^{I}\tau_{i}v_{i}^{*}\leq 1$, it is less than the optimal value of the Model (20), that is $\Theta_{p}^{\xi *}\sum_{i=1}^{I}\tau_{i}v_{i}^{*}\leq \Theta_{p}^{\xi *}$, we will have $\Theta_{p}^{\xi *}\sum_{i=1}^{I}\tau_{i}v_{i}^{*}\leq \Theta_{p}^{\xi *}\leq \sum_{t=1}^{T}\mu_{t}w_{t}^{*}$, as a result, the fifth constraint is established. Now we have $\sum_{t=1}^{T}\rho_{t}w_{t}^{*}\leq \sum_{i=1}^{I}\gamma_{i}v_{i}^{*}$ from Model (20), if the constraint is $\sum_{t=1}^{T}\rho_{t}w_{t}^{*}\leq 1$, then the second constraint is established and the Proposition is proofed. Otherwise, suppose $\sum_{t=1}^{T}\rho_{t}w_{t}^{*}=c>1$ (*c* is a constant number), let the items be replaced in the following manner: ${\bar{v}}_{i}=\frac{1}{c}v_{t}^{*}$, ${\bar{w}}_{t}=\frac{1}{c}w_{t}^{*}$, ${\bar{u}}_{r}=\frac{1}{c}\varepsilon$, and Υ = 0, as a result, $\sum_{t=1}^{T}\rho_{t}{\bar{w}}_{t}=\frac{1}{c}\sum_{t=1}^{T}\rho_{t}w_{t}^{*}=1\leq 1$ and the second constraint is established. As the rest of the constraints were established before, so even if they are multiplied by $\frac{1}{c}>0$, they are still established and the Proposition is proofed. Accordinlgy, for Model (21), there exists a feasible solution.

Finally, the overall efficiency score under specific confidence level *ξ* is calculated according to Eq (22) as follows:

$$\Psi_{p}^{\xi *}=\Theta_{p}^{\xi *}\;\Phi_{p}^{\xi *}$$ (22)

In an alternative scenario, considering the possibility that second stage holds greater significance (second stage is considered as leader), in a similar manner, the efficiency score of the second stage under Z-information is calculated at first and then the efficiency score of first stage will be calculated. Finally, the efficiency score of the whole of two-stage system will be measured using Eq (22).

## 5. Case study: Iranian private insurance companies

**Fig 2:**
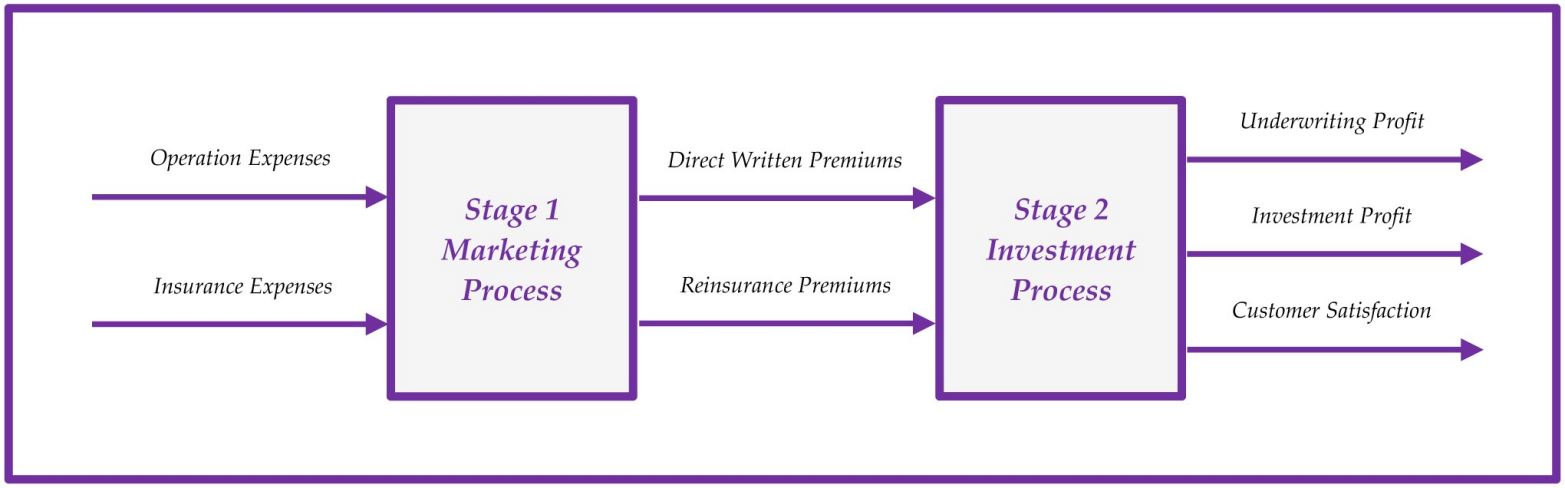
The network structure of insurance company.

In this section, the proposed Z-number NDEA approach will be applied to measure the performance of Iranian private insurance companies (IPICs) in Iran. It’s important to acknowledge that insurance companies play a vital role as one of the key foundations of the financial markets. As a result, many researches have focused on performance assessment of insurance companies using network DEA approach [29–33]. It should be explained that the internal structure of insurance companies can be considered as a two-stage system including the marketing and investment processes [29]. Fig 2 graphically illustrates a two-stage network structure of insurance companies:

The explanations for the variables introduced in Fig 2 are as follows. **Operation Costs (OC)**: *Refers to the expenditures related to employee salaries and various expenses that are incurred in the course of daily work operations*. **Insurance Costs (IC)**: *Encompasses the expenses incurred in marketing insurance services*, *including payments made to agencies*, *brokers*, *lawyers*, *and other associated costs*. **Direct Written Premium (DWP)**: *Denotes the premium received from customers who have purchased insurance coverage*. **Reinsurance Premium (RP)**: *Represents the premium received from companies involved in transferring insurance risks*. **Underwriting Profit (UP)**: *Signifies the profit generated from the insurance business itself*, *taking into account the costs and risks associated with underwriting policies*. **Investment Profit (IP)**: *Represents the profit gained from the insurance company’s investment portfolio*. **Customer Satisfaction (CS)**: *Refers to the level of satisfaction expressed by customers*, *which is measured as a linguistic variable*. Now, the Z-number data for 14 Iranian insurance companies are extracted. Data set for inputs, intermediate measures, and outputs are presented in Tables 2 to 4, respectively:

**Table 2:** The data set for inputs of Iranian private insurance companies.

IPICs	Operation Expenses (X_1_)	Insurance Expenses (X_2_)
IPIC 01	(49322, 52062, 57542, 60282; U)	(360454, 380479, 420529, 440554; L)
IPIC 02	(3545, 3742, 4136, 4333; S)	(114863, 121244, 134006, 140388; L)
IPIC 03	(4278, 4515, 4991, 5228; L)	(37601, 39690, 43868, 45957; S)
IPIC 04	(75533, 79729, 88121, 92318; U)	(707839, 747164, 825812, 865137; L)
IPIC 05	(38562, 40705, 44989, 47132; L)	(1046359, 1104490, 1220752, 1278883; U)
IPIC 06	(24931, 26316, 29086, 30471; S)	(144383, 152404, 168446, 176468; U)
IPIC 07	(22174, 23406, 25870, 27102; U)	(60494, 63855, 70577, 73938; S)
IPIC 08	(1193, 1260, 1392, 1459; L)	(9068, 9571, 10579, 11083; L)
IPIC 09	(34850, 36786, 40658, 42594; L)	(2247380, 2372235, 2621943, 2746798; S)
IPIC 10	(9702, 10241, 11319, 11858; S)	(61213, 64613, 71415, 74815; S)
IPIC 11	(17043, 17990, 19884, 20831; L)	(452163, 477283, 527523, 552643; U)
IPIC 12	(23027, 24306, 26864, 28144; L)	(161146, 170098, 188004, 196956; L)
IPIC 13	(39434, 41625, 46007, 48198; L)	(662704, 699521, 773155, 809972; U)
IPIC 14	(12556, 13253, 14649, 15346; U)	(37563, 39650, 43824, 45911; U)

**Table 3:** The data set for intermediate measures of Iranian private insurance companies.

IPICs	Direct Written Premiums (Z_1_)	Reinsurance Premiums (Z_2_)
IPIC 01	(391908, 413680, 457226, 478998; U)	(75784, 79994, 88414, 92624; U)
IPIC 02	(115853, 122289, 135161, 141598; S)	(3758, 3967, 4385, 4594; L)
IPIC 03	(58362, 61605, 68089, 71332; U)	(2847, 3005, 3321, 3479; U)
IPIC 04	(794566, 838708, 926994, 971136; L)	(74849, 79008, 87324, 91483; L)
IPIC 05	(1288443, 1360023, 1503183, 1574763; L)	(124845, 131781, 145653, 152589; S)
IPIC 06	(200664, 211812, 234108, 245256; S)	(19631, 20721, 22903, 23993; U)
IPIC 07	(150971, 159358, 176132, 184520; S)	(14494, 15299, 16909, 17714; U)
IPIC 08	(11792, 12447, 13757, 14412; U)	(3691, 3896, 4306, 4511; L)
IPIC 09	(1346245, 1421037, 1570619, 1645411; U)	(235245, 248314, 274452, 287521; L)
IPIC 10	(88322, 93228, 103042, 107949; L)	(665, 702, 776, 813; S)
IPIC 11	(553714, 584476, 646000, 676762; L)	(55724, 58820, 65012, 68108; U)
IPIC 12	(222358, 234711, 259417, 271770; U)	(5040, 5320, 5880, 6160; L)
IPIC 13	(711991, 751546, 830656, 870211; S)	(94829, 100098, 110634, 115903; U)
IPIC 14	(65469, 69106, 76380, 80017; L)	(2457, 2594, 2867, 3003; S)

**Table 4:** The data set for outputs of Iranian private insurance companies.

IPICs	Underwriting Profit (Y_1_)	Investment Profit (Y_2_)	Customer Satisfaction (Y_3_)
IPIC 01	(73502, 77586, 85752, 89836; L)	(14302, 15096, 16686, 17480; S)	(M; U)
IPIC 02	(3744, 3952, 4368, 4576; S)	(2490, 2629, 2905, 3044; L)	(VL; U)
IPIC 03	(17880, 18874, 20860, 21854; L)	(11638, 12284, 13578, 14224; S)	(M; L)
IPIC 04	(98993, 104492, 115492, 120991; L)	(27667, 29204, 32278, 33815; L)	(M; U)
IPIC 05	(66601, 70301, 77701, 81401; L)	(152665, 161147, 178109, 186591; U)	(VH; L)
IPIC 06	(15837, 16717, 18477, 19357; S)	(27409, 28931, 31977, 33499; L)	(MH; L)
IPIC 07	(28059, 29618, 32736, 34295; U)	(22174, 23406, 25870, 27102; U)	(ML; U)
IPIC 08	(1128, 1190, 1316, 1378; L)	(2470, 2607, 2881, 3018; S)	(L; L)
IPIC 09	(192602, 203302, 224702, 235402; L)	(69022, 72856, 80526, 84360; L)	(MH; U)
IPIC 10	(27874, 29422, 32520, 34068; U)	(44609, 47088, 52044, 54523; U)	(H; L)
IPIC 11	(938096, 40213, 44445, 46562; S)	(57209, 60388, 66744, 69923; L)	(H; U)
IPIC 12	(57521, 60716, 67108, 70303; L)	(10613, 11202, 12382, 12971; S)	(M; U)
IPIC 13	(68783, 72604, 80246, 84068; L)	(20380, 21512, 23776, 24908; L)	(ML; L)
IPIC 14	(12817, 13529, 14953, 15665; U)	(10954, 11562, 12780, 13388; U)	(M; U)

Please note that Table 5 presents an equivalent fuzzy number for linguistic variables and reliability components of Z-numbers as follows:

**Table 5:** The linguistic variables and their associated fuzzy number in this study.

First Term of Z-Number		Second Term of Z-Number	
Linguistic Variable	Trapezoidal Fuzzy Number	Linguistic Variable	Triangular Fuzzy Number
Vl: Very Low	(0.10, 0.20, 0.30, 0.40)	L: Likely	(0.50, 0.60, 0.70)
L: Low	(0.20, 0.30, 0.40, 0.50)	U: Usually	(0.65, 0.75, 0.85)
ML: Medium Low	(0.30, 0.40, 0.50, 0.60)	S: Sure	(0.80, 1.00, 1.00)
M: Medium	(0.40, 0.50, 0.60, 0.70)		
MH: Medium High	(0.50, 0.60, 0.70, 0.80)		
H: High	(0.60, 0.70, 0.80, 0.90)		
VH: Very High	(0.70, 0.80, 0.09, 1.00)		

Now, after collecting data in Z-number, using method of Kang et al. [77], all Z-number will be converted to classic fuzzy numbers with trapezoidal membership function. Then, the proposed ZNDEA models will be run for confidence levels to measure the efficiency scores of stage 1, stage 2, and whole of system. Accordingly, Table 6 presents the results of ZNDEA approach when the stage 1 (marketing process) is more important. Also, Table 7 introduces the results of proposed approach when the stage 2 (investment process) is more important.

**Table 6:** The results of ZNDEA approach—First stage is a leader.

	IPICs	Confidence Levels				
		60%	70%	80%	90%	100%
**Marketing Process (Stage 1)**	IPIC 01	0.59226	0.56892	0.54648	0.52488	0.50410
	IPIC 02	0.78639	0.75543	0.72565	0.69699	0.66944
	IPIC 03	0.73986	0.71073	0.68270	0.65574	0.62980
	IPIC 04	0.56510	0.54285	0.52144	0.50084	0.48103
	IPIC 05	0.78640	0.75544	0.72565	0.69699	0.66942
	IPIC 06	0.64431	0.61894	0.59454	0.57106	0.54846
	IPIC 07	0.78639	0.75543	0.72565	0.69699	0.66942
	IPIC 08	0.78685	0.75568	0.72581	0.69714	0.66955
	IPIC 09	0.78640	0.75544	0.72566	0.69701	0.66944
	IPIC 10	0.51400	0.49376	0.47429	0.45556	0.43754
	IPIC 11	0.77218	0.74178	0.71253	0.68439	0.65732
	IPIC 12	0.71223	0.68418	0.65721	0.63125	0.60628
	IPIC 13	0.76956	0.73925	0.71009	0.68204	0.65505
	IPIC 14	0.49122	0.47188	0.45328	0.43538	0.41815
**Investment Process (Stage 2)**	IPIC 01	0.33482	0.32164	0.30897	0.29678	0.28505
	IPIC 02	0.09022	0.08181	0.07421	0.06732	0.06196
	IPIC 03	0.62285	0.58881	0.55707	0.52812	0.50723
	IPIC 04	0.26360	0.25313	0.24316	0.23356	0.22432
	IPIC 05	0.18378	0.17657	0.15067	0.14713	0.13896
	IPIC 06	0.17966	0.17025	0.16146	0.15322	0.14549
	IPIC 07	0.37132	0.35670	0.27209	0.24894	0.22916
	IPIC 08	0.63620	0.57058	0.51189	0.44690	0.41151
	IPIC 09	0.28519	0.27396	0.26316	0.25277	0.24277
	IPIC 10	0.78640	0.75544	0.72566	0.69700	0.66943
	IPIC 11	0.17141	0.16719	0.15970	0.15290	0.14661
	IPIC 12	0.51567	0.49537	0.47584	0.45704	0.43896
	IPIC 13	0.15323	0.14705	0.14119	0.13559	0.13023
	IPIC 14	0.55691	0.52587	0.49698	0.47008	0.44492
**Insurance Companies (Overall)**	IPIC 01	0.19830	0.18299	0.16885	0.15578	0.14370
	IPIC 02	0.07095	0.06180	0.05385	0.04692	0.04148
	IPIC 03	0.46082	0.41848	0.38032	0.34631	0.31946
	IPIC 04	0.14896	0.13741	0.12679	0.11698	0.10791
	IPIC 05	0.14453	0.13339	0.10934	0.10255	0.09302
	IPIC 06	0.11576	0.10538	0.09599	0.08750	0.07979
	IPIC 07	0.29200	0.26946	0.19744	0.17351	0.15340
	IPIC 08	0.50059	0.43118	0.37154	0.31155	0.27553
	IPIC 09	0.22427	0.20696	0.19096	0.17618	0.16252
	IPIC 10	0.40421	0.37301	0.34418	0.31753	0.29290
	IPIC 11	0.13236	0.12402	0.11379	0.10465	0.09637
	IPIC 12	0.36727	0.33892	0.31272	0.28851	0.26613
	IPIC 13	0.11792	0.10871	0.10026	0.09248	0.08531
	IPIC 14	0.27356	0.24815	0.22527	0.20466	0.18605

**Table 7:** The results of ZNDEA approach—Second stage is a leader.

	IPICs	Confidence Levels				
		60%	70%	80%	90%	100%
**Marketing Process (Stage 1)**	IPIC 01	0.54723	0.52597	0.50613	0.48552	0.46621
	IPIC 02	0.73229	0.71923	0.70769	0.69699	0.66944
	IPIC 03	0.73790	0.70886	0.68094	0.65400	0.62821
	IPIC 04	0.55045	0.52883	0.50844	0.48826	0.46921
	IPIC 05	0.78532	0.75365	0.72401	0.69633	0.66740
	IPIC 06	0.63676	0.61243	0.58747	0.56506	0.54195
	IPIC 07	0.78639	0.75543	0.72564	0.69699	0.66941
	IPIC 08	0.69184	0.66729	0.64040	0.61308	0.58963
	IPIC 09	0.78640	0.75544	0.72566	0.69701	0.66944
	IPIC 10	0.51400	0.49376	0.47429	0.45556	0.43754
	IPIC 11	0.76586	0.73712	0.70744	0.67966	0.65334
	IPIC 12	0.71223	0.68418	0.65721	0.63125	0.60628
	IPIC 13	0.71566	0.68824	0.65943	0.63248	0.61059
	IPIC 14	0.48558	0.47188	0.45328	0.43538	0.41815
**Investment Process (Stage 2)**	IPIC 01	0.37387	0.35915	0.34498	0.33136	0.31825
	IPIC 02	0.11418	0.09741	0.08205	0.06797	0.06196
	IPIC 03	0.63329	0.60040	0.56960	0.54129	0.51988
	IPIC 04	0.27767	0.26674	0.25622	0.24610	0.23637
	IPIC 05	0.18448	0.17722	0.17023	0.16351	0.15704
	IPIC 06	0.18624	0.17690	0.16815	0.15991	0.15215
	IPIC 07	0.37132	0.35670	0.34264	0.32911	0.31609
	IPIC 08	0.65017	0.58779	0.53176	0.48125	0.43555
	IPIC 09	0.28519	0.27396	0.26316	0.25277	0.24277
	IPIC 10	0.78640	0.75544	0.72591	0.69752	0.67019
	IPIC 11	0.19125	0.18372	0.17647	0.16950	0.16280
	IPIC 12	0.51567	0.49537	0.47584	0.45704	0.43896
	IPIC 13	0.17263	0.16583	0.15930	0.15301	0.14695
	IPIC 14	0.58267	0.53114	0.49698	0.47008	0.44492
**Insurance Companies (Overall)**	IPIC 01	0.20459	0.18890	0.17461	0.16088	0.14837
	IPIC 02	0.08362	0.07006	0.05806	0.04737	0.04148
	IPIC 03	0.46731	0.42560	0.38786	0.35400	0.32659
	IPIC 04	0.15284	0.14106	0.13027	0.12016	0.11090
	IPIC 05	0.14488	0.13356	0.12325	0.11386	0.10481
	IPIC 06	0.11859	0.10834	0.09878	0.09036	0.08246
	IPIC 07	0.29200	0.26946	0.24863	0.22938	0.21159
	IPIC 08	0.44981	0.39223	0.34054	0.29505	0.25682
	IPIC 09	0.22427	0.20696	0.19096	0.17618	0.16252
	IPIC 10	0.40421	0.37301	0.34429	0.31776	0.29324
	IPIC 11	0.14647	0.13542	0.12484	0.11520	0.10636
	IPIC 12	0.36727	0.33892	0.31272	0.28851	0.26613
	IPIC 13	0.12355	0.11413	0.10504	0.09677	0.08973
	IPIC 14	0.28293	0.25063	0.22527	0.20466	0.18605

**Fig 3:**
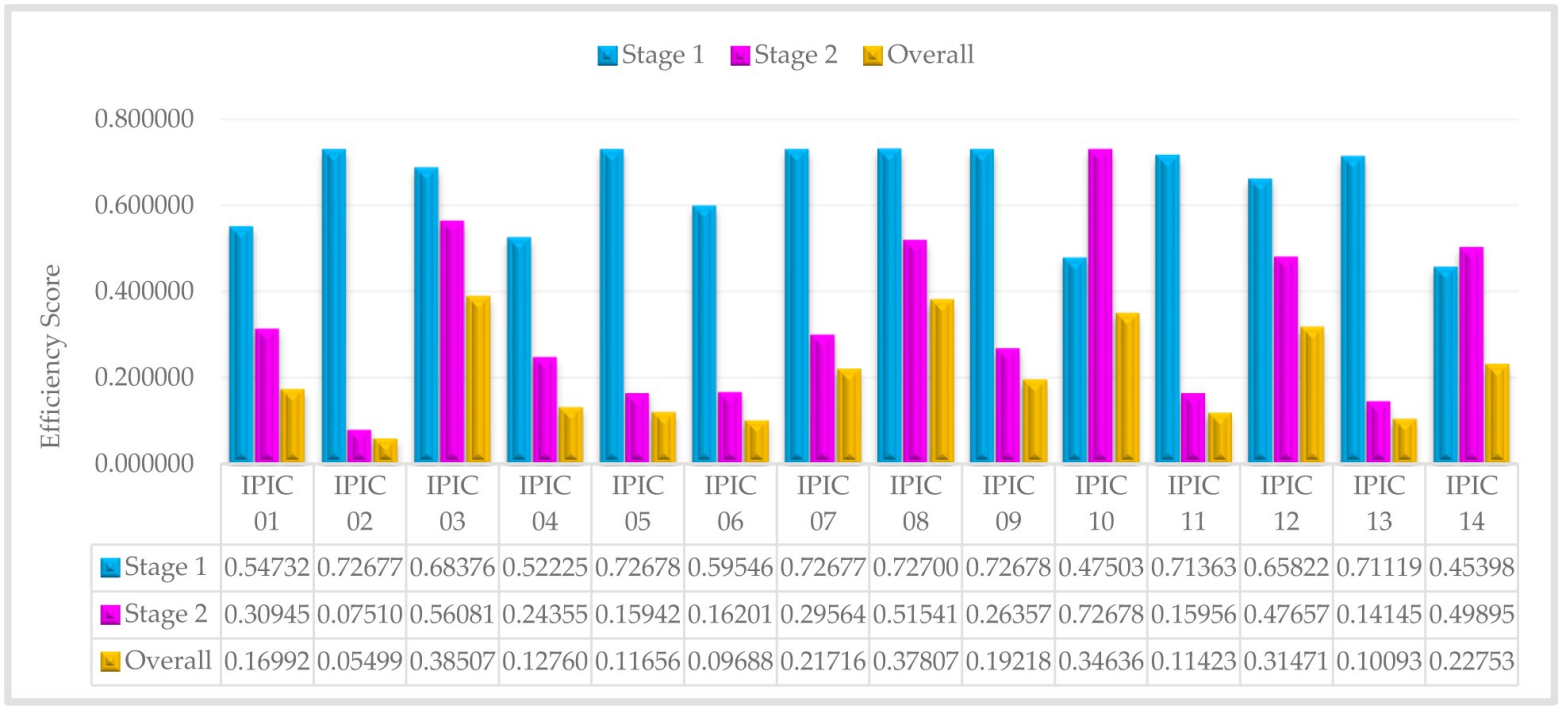
The average efficiency score of IPICs—Stage 1 is a leader.

**Fig 4:**
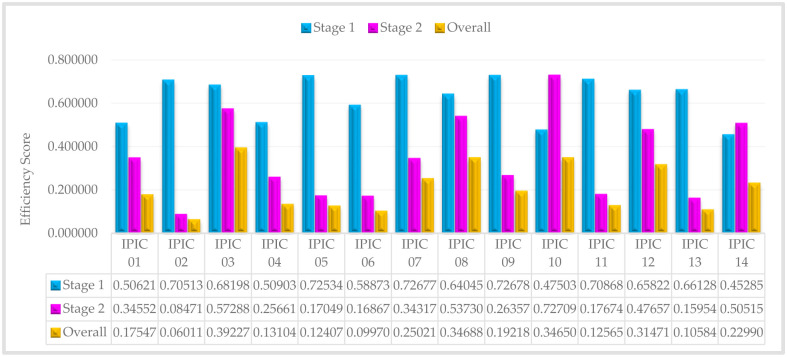
The average efficiency score of IPICs—Stage 2 is a leader.

**Fig 5:**
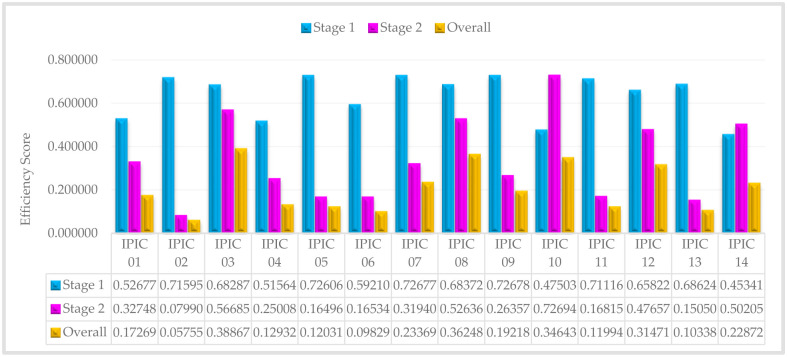
The final score and ranking of IPICs based on ZNDEA approach.

The efficiency scores of IPICs and sub-IPICs decrease as the confidence levels increase from 60% to 100%, as evident from Tables 6 and 7. Now, in order to evaluate and rank insurance companies, we compute the average efficiency score for each company across all confidence levels. Accordingly, the average efficiency for situation that first stage or second stage is leader, are introduced in Figs 3 and 4, respectively:

In the end, an average efficiency score is computed for both instances of leadership in the initial and subsequent stages. The ranking of all 14 Iranian private insurance companies based on ZNDEA approach is given in Fig 5:

In Fig 5, it is evident that IPIC 09 and IPIC 10 exhibit exemplary performance in the marketing process and investment process, respectively. Additionally, when compared to other private insurance companies, IPIC 03 stands out with the highest overall performance. Consequently, these private insurance companies serve as exemplars or benchmarks for other private insurance company managers to emulate. Essentially, the management practices and strategies employed by these top-ranking companies can be adopted by other private insurance companies seeking to enhance their performance and efficiency. By studying and implementing the successful approaches of these leading private insurance companies, other managers can strive to achieve similar levels of excellence and success in their own operations.

As previously elucidated in the modeling process, the overall efficiency of the whole system is determined by multiplying the efficiencies of both the initial and subsequent stages. Consequently, any subpar performance in individual sub-processes significantly impacts the efficiency of the entire system, resulting in an overall decline in system performance. This interconnected relationship highlights the critical importance of maintaining high performance levels across all stages to ensure optimal system efficiency. For example, IPIC 02, despite showcasing strong performance in the initial stage (0.71595), ultimately displays the lowest overall performance (0.05755) among all Iranian private insurance companies. This can be attributed to its notably low and unfavorable performance in the subsequent stage (0.07990). Therefore, despite an initially positive performance, the substandard performance in the second stage significantly affects IPIC 02’s overall ranking within the Iranian private insurance industry.

Therefore, to enhance the efficiency of systems exhibiting undesirable performance, it is crucial to identify the stage responsible for inefficiencies. Subsequently, benchmarks can be established using the performance metrics of IPIC 09 (0.72678) and IPIC 10 (0.72694) for the first and second stages, respectively. This approach enables targeted efforts to improve the performance of the sub-DMUs, thereby leading to enhancements in the overall system efficiency.

It should be explained that the adoption of the ZNDEA method for performance assessment of insurance companies under data uncertainty and network structure holds significant economic and managerial implications for the insurance industry. By leveraging this approach, insurance companies can enhance their performance evaluation, leading to more accurate assessments of operational efficiency and effectiveness. This, in turn, can improve decision-making processes, resource management, and strategic planning, ultimately boosting the overall performance of insurance firms. Moreover, the methodology’s capability to handle data uncertainty and network structures can enhance risk management practices within insurance companies. This robust framework for risk assessment enables insurers to identify and mitigate potential risks more effectively, promoting better financial stability and resilience in the face of uncertainties.

Additionally, insights derived from this analysis can aid policymakers in developing targeted regulatory interventions that foster fair competition and consumer protection. This can enhance market transparency, integrity, and efficiency, creating a more competitive landscape within the insurance industry. Furthermore, the adoption of advanced analytical approaches like the ZNDEA can drive innovation within the insurance sector. Companies may invest in technology and data analytics to enhance performance assessment processes, leading to industry-wide advancements in operational efficiency and customer service. Lastly, the network analysis aspect of this approach can facilitate the identification of strategic partnerships and collaboration opportunities within the insurance sector. By optimizing relationships with stakeholders and enhancing information sharing, companies can create synergies that benefit the industry as a whole. In summary, implementing the Z-number network DEA approach in the insurance sector can contribute to long-term sustainability and growth in a dynamic and uncertain business environment.

## 6. Conclusions and future research directions

Performance evaluation of homogeneous DMUs with the aim of identifying efficient units and benchmarking for improving inefficient units, is always one of the most widely used areas in both theoretical and real-world problems. One of the most widely used and powerful approaches in the field of performance measurement is the data envelopment analysis approach. In the current paper, a new uncertain network DEA (UNDEA) approach is proposed that is capable to be utilized for performance assessment of DMUs with two-stage network structure under Z-information. It should be explained that to propose a novel UNDEA approach, the leader-follower approach for NDEA modeling as well as Z-number theory and credibility measure for handling data ambiguity are applied. Also, to demonstrate the practical application and effectiveness of the proposed ZNDEA method, a genuine dataset was employed to assess the efficiency of 14 Iranian private insurance companies. Notably, the research on the ZNDEA approach for performance evaluation of insurance companies provides a systematic framework for policymakers to evaluate the efficiency and effectiveness of insurance firms amidst data uncertainty.

By leveraging this methodology, policymakers can gain a comprehensive understanding of the operational performance of insurance companies, enabling them to identify areas for improvement and implement targeted interventions. This approach offers a more nuanced perspective on performance assessment by considering various factors that may impact the decision-making process, such as ambiguity and imprecision in data. Moreover, by incorporating ZNDEA results into policymaking strategies, regulators can enhance their ability to monitor and supervise insurance companies, thereby promoting market stability and consumer protection. The application of this approach can lead to more informed regulatory decisions, improved risk management practices, and a more competitive insurance market. Ultimately, the integration of the findings from this research into policymaking initiatives can contribute to the overall resilience, efficiency, and sustainability of the insurance sector, benefiting both industry stakeholders and consumers alike.

The main advantages of the proposed ZNDEA approach can be succinctly outlined as follows: The presented ZNDEA approach is capable of being utilized within Z-information. It is also designed to function effectively within a two-stage network structure. The ZNDEA method offers an efficient means of ranking two-stage network DMUs. This approach is formulated in a linear programming framework. Notably, the efficiency decomposition is distinct due to the proposed ZNDEA method. It demonstrates the capability to be implemented in the presence of financial data characterized by deep uncertainty. It allows for a comprehensive assessment of DMUs across various scenarios, determining the significance of each stage. Moreover, the proposed ZNDEA method enhances the discriminatory power of results and increases result reliability through the application of Z-information. Additionally, it helps identify the sensitivity and stability levels of DMUs towards data uncertainty. Finally, the ZNDEA approach can be expanded to accommodate an extended two-stage and series structures.

In terms of the limitations of the proposed ZNDEA approach regarding modeling and theory, the simplification and conversion of Z numbers to classical fuzzy numbers can be mentioned. Additionally, from a practical standpoint, a significant limitation of utilizing the Z-number network DEA approach to evaluate the performance of insurance companies is the potential oversight of external factors and the dynamic nature of the industry. These external variables, such as governmental policy changes, shifts in insurance regulations, economic fluctuations, and advancements in insurance technology, are diverse and can significantly impact performance. Failing to consider these external influences may result in an incomplete understanding of the factors affecting insurance companies’ performance, potentially restricting the applicability and robustness of evaluation outcomes in real-world settings. Therefore, integrating these external dynamics into the ZNDEA analysis could offer a more comprehensive and insightful evaluation of insurance company performance within a broader framework.

Although the research offers valuable insights into assessing the performance of insurance companies under conditions of data uncertainty and network structure, the omission of external factors in the analysis poses a limitation that may affect the overall strength and applicability of the results. Future studies could overcome this limitation by embracing a more comprehensive approach that considers the broader contextual factors influencing the performance of insurance companies in uncertain environments. Alos, for the future studies, the other popular and powerful uncertain programming approaches such as robust optimization [82–87], uncertainty theory [88–92], interval programming [93–99], and stochastic optimization [100–105], can also be employed to deal with different type of data uncertainty.
